# Beyond the Thrombus: Imaging‐Guided Management of Acute Coronary Syndrome

**DOI:** 10.1002/ccr3.72652

**Published:** 2026-05-04

**Authors:** Guillaume Guebey, Mathieu Chong, Adelin Barrier, Sarah Mauler‐Wittwer, Stéphane Noble, Quentin Liabot

**Affiliations:** ^1^ Cardiology Department Geneva University Hospitals Geneva Switzerland; ^2^ Interventional Cardiology Unit Geneva University Hospitals Geneva Switzerland; ^3^ Structural Cardiology Unit Geneva University Hospitals Geneva Switzerland

**Keywords:** acute coronary syndrome, imaging‐guided percutaneous coronary intervention, intracoronary imaging, optical coherence tomography, percutaneous coronary intervention, plaque rupture

## Abstract

Intracoronary imaging is a true game‐changer in acute coronary syndromes, enhancing diagnostic accuracy while ensuring precise, mechanism‐driven, and patient‐tailored management.

## Graphical abstract

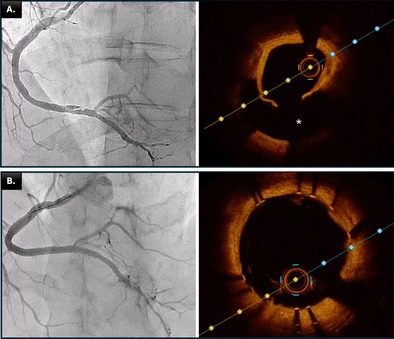

## 1 Introduction

While the benefit of revascularization in chronic coronary syndromes remains a matter of ongoing debate [1], it represents the gold standard for the management of acute coronary syndromes (ACS) [2]. However, angiography alone provides limited information regarding the underlying mechanisms of ACS. Intracoronary imaging, notably optical coherence tomography (OCT), offers high‐resolution characterization of coronary plaques, enabling differentiation between plaque rupture, erosion, calcified nodule, or spontaneous dissection, the main mechanisms of ACS. Accurate management hinges on identifying these underlying lesion morphologies [3] to guide optimal treatment. We present the case of a young patient with ACS in whom OCT played a pivotal role, enabling precise and tailored management.

## 2 Case History/Examination

A 55‐year‐old male without cardiovascular risk factors presented to the emergency department several hours after experiencing acute, oppressive chest pain associated with profuse sweating during a football match. By the time of presentation, his symptoms had resolved. The electrocardiogram (ECG) demonstrated a regular sinus rhythm without repolarization disturbances, but high‐sensitivity troponins were elevated, peaking at 496 ng/L (normal < 14 ng/L). Transthoracic echocardiography (TTE) revealed a normal left ventricular ejection fraction with no wall‐motion abnormalities. Accordingly, the patient received an intravenous loading dose of aspirin (250 mg) together with fondaparinux (2.5 mg) in the context of suspected non‐ST‐segment elevation myocardial infarction (NSTEMI).

## 3 Differential Diagnosis, Investigations and Treatment

At this stage, no credible alternative differential diagnosis was considered, as the clinical presentation and troponin elevation were highly suggestive of NSTEMI, warranting prompt coronary angiography.

**FIGURE 1:**
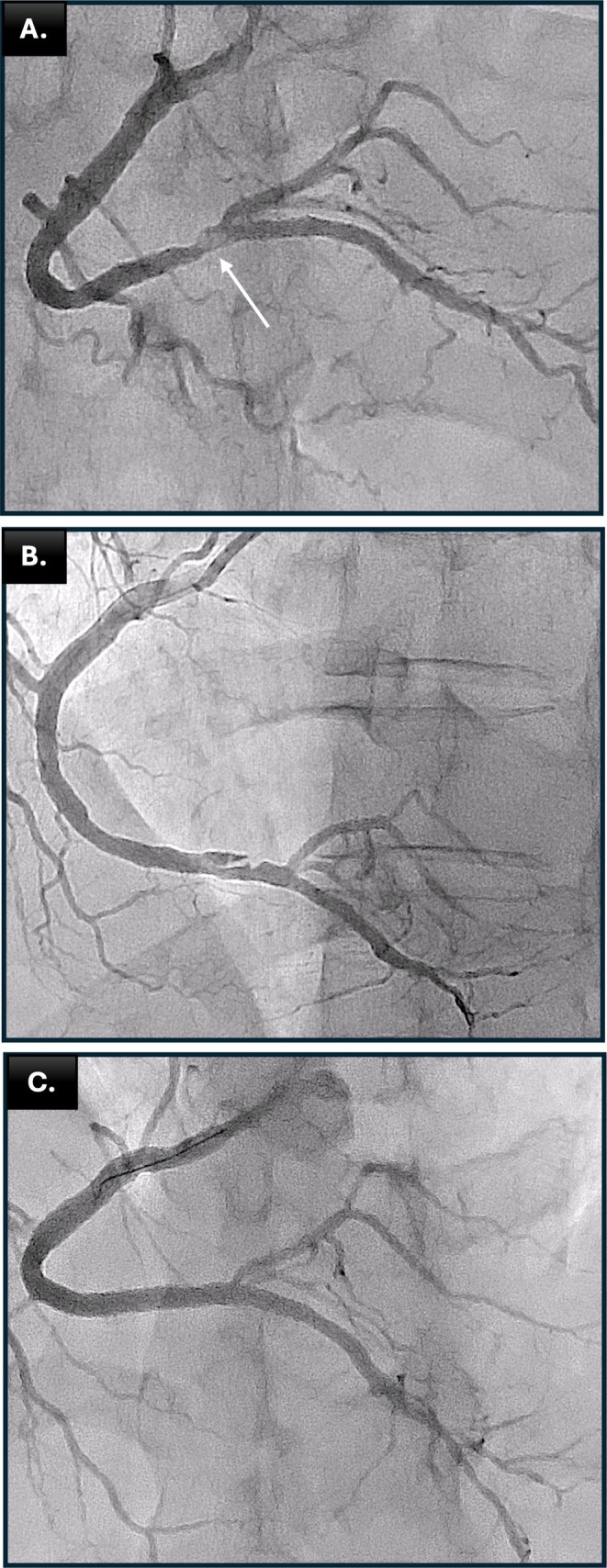
Coronary Angiography Findings. (A) Thrombotic appearance of the distal RCA (white arrow), at the bifurcation of the posterolateral branch and the posterior descending artery. (B) Angiographic appearance consistent with plaque rupture on one‐month follow‐up angiography. (C) Angiographic result after stenting.

Coronary angiography was performed within the first 24 h and revealed single‐vessel disease characterized by a markedly thrombotic appearance of the distal right coronary artery (RCA), at the bifurcation of the posterolateral branch and the posterior descending artery (PDA) (Figure 1A, Video 1), without significant lesion in the left coronary system. Given the preserved flow (TIMI 3), absence of ongoing chest pain, and the risk of distal embolization or occlusion of one of the two distal branches, the lesion was left untreated. Consequently, conservative management was initiated with triple antithrombotic therapy (aspirin, clopidogrel, plus a direct oral anticoagulant). The patient was discharged home after a few uneventful days of monitoring, with a follow‐up coronary angiography scheduled 1 month later, along with sick leave and physical activity restriction.

**VIDEO 1:** Thrombotic appearance of the distal RCA at the bifurcation of the posterolateral and posterior descending arteries. Video content can be viewed at https://onlinelibrary.wiley.com/doi/10.1002/ccr3.72652.

**FIGURE 2:**
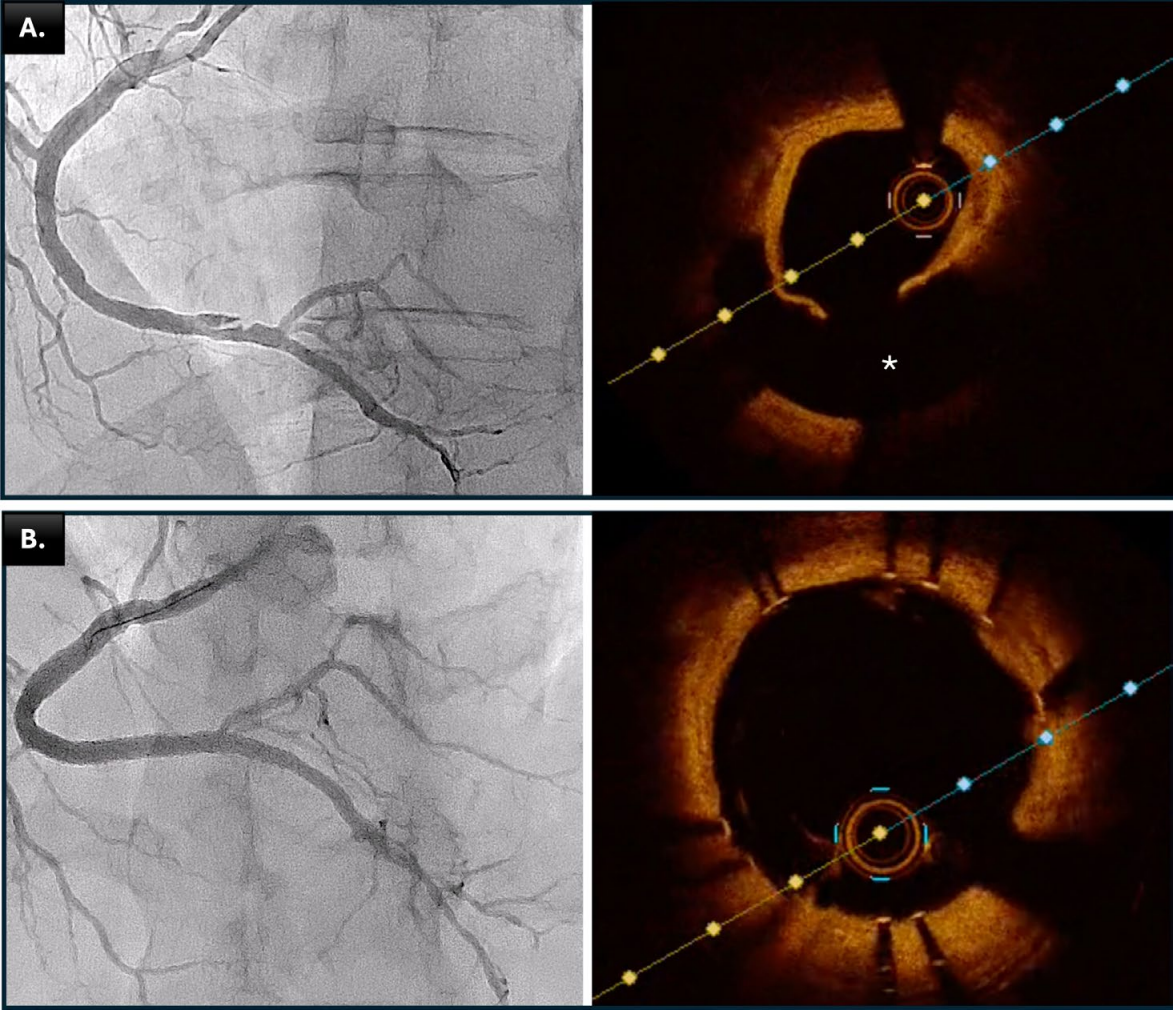
Intracoronary Imaging Assessment. (A) Plaque rupture with disruption of the lipid core and a large, wide intimal breach on OCT (*) at one‐month follow‐up angiography. (B) Final OCT result with optimal stent apposition.

At 1 month, angiography revealed complete thrombus clearance with an angiographic appearance consistent with plaque rupture (Figure 1B, Video 2). OCT confirmed plaque rupture, with disruption of the lipid core and a large, wide intimal breach (Figure 2A, Video 3). Given the plaque instability and the underlying stenosis, the patient underwent OCT‐guided percutaneous coronary intervention (PCI) with implantation of a sirolimus‐eluting stent (3.5 × 28 mm, Ultimaster Nagomi, Terumo) in the distal RCA, extending toward the PDA, to fully cover the atherosclerotic lesion. Immediate angiographic and OCT results were excellent, with optimal stent apposition (Figures 1C and 2B, Videos 4 and 5).

**VIDEO 2:** Complete thrombus resolution with angiographic features consistent with plaque rupture at 1‐month follow‐up angiography. Video content can be viewed at https://onlinelibrary.wiley.com/doi/10.1002/ccr3.72652.

**VIDEO 3:** Plaque rupture on OCT with disruption of the lipid core and a large, wide intimal breach. Video content can be viewed at https://onlinelibrary.wiley.com/doi/10.1002/ccr3.72652.

**VIDEO 4:** Excellent immediate angiographic result after stenting. Video content can be viewed at https://onlinelibrary.wiley.com/doi/10.1002/ccr3.72652.

**VIDEO 5:** OCT result with optimal stent apposition. Video content can be viewed at https://onlinelibrary.wiley.com/doi/10.1002/ccr3.72652.

## 4 Conclusion and Results

The patient's in‐hospital course was uncomplicated, with no adverse events reported. Both ECG and TTE remained stable, with no new abnormalities.

After one‐month follow‐up angiography, the antithrombotic regimen was de‐escalated to dual antiplatelet therapy with aspirin and prasugrel, planned for a standard 12‐month duration. At 6 months, the patient was still in excellent clinical condition, asymptomatic, and free of any cardiac or systemic complications.

## 5 Discussion

Although current guidelines [2] do not recommend deferring stent implantation in the context of ACS, specific clinical scenarios may justify a delayed approach. In carefully selected patients, such as the one presented here, with preserved flow in the infarct‐related artery, no ST‐segment elevation, and no ongoing chest pain, postponing stenting can allow for a more accurate assessment of the underlying lesion and facilitate an imaging‐guided, patient‐tailored strategy.

The initial decision to avoid immediate stenting was driven by the large thrombotic burden involving the distal RCA bifurcation between the posterolateral and posterior descending arteries. In such a setting, immediate stent implantation could have carried a significant risk of distal embolization or inadvertent occlusion of one of the two branches.

Furthermore, plaque erosion was initially considered a plausible diagnosis, as it is a frequent mechanism of NSTEMI in young patients [3, 4]. Although plaque rupture remains the most common mechanism overall, plaque erosion represents a highly relevant differential diagnosis in young patients with few cardiovascular risk factors. Previous data suggest that, in selected patients with plaque erosion and no underlying significant stenosis, conservative antithrombotic therapy without stenting may lead to satisfactory vessel healing [5]. Therefore, in a clinically stable patient (pain‐free, with no ECG changes and preserved flow in the infarct‐related artery), this diagnostic uncertainty provided an additional rationale to avoid premature stent implantation. This rationale supported the initial choice of triple antithrombotic therapy to promote thrombus resolution before delayed reassessment.

Performing OCT during the acute phase in the presence of a large thrombus burden offers limited diagnostic accuracy, as the thrombus may obscure the underlying plaque morphology. In such situations, delayed imaging after adequate thrombus clearance provides a more reliable assessment of the culprit lesion and ensures an appropriate treatment strategy [6]. Importantly, when the initial thrombotic burden is substantial, very early reassessment within the first few days may still be hampered by persistent thrombus, thereby limiting the additional diagnostic value of intracoronary imaging. A longer interval allowing more complete thrombus resolution may therefore be safe and reasonable, as illustrated in the present case.

This case also raised the question of a potential cardio‐embolic etiology. OCT was instrumental in ruling out this hypothesis by demonstrating a clear plaque complication, thereby avoiding an extensive and costly work‐up for an emboligenic cardiopathy that would have been unnecessary. Spontaneous coronary artery dissection (SCAD) should also be considered in young patients presenting with ACS and few cardiovascular risk factors. However, SCAD predominantly affects women and typically occurs in the absence of significant atherosclerotic disease and is associated with specific angiographic patterns, which were not observed in the present case [7]. Eventually, follow‐up OCT demonstrated a plaque rupture, further excluding this hypothesis.

The potential role of aspiration thrombectomy also warrants discussion. Randomized trials have consistently failed to demonstrate a clinical benefit of routine thromboaspiration [8], and concerns remain regarding the risk of cerebral or systemic embolization. In this specific context, with preserved TIMI 3 flow, normal ECG, and no residual chest pain, the benefit of thrombectomy was uncertain and the potential associated risks non‐negligible, supporting the decision not to perform it. Similarly, with respect to glycoprotein IIb/IIIa inhibitors, the absence of expected ischemic benefit and the potential risk of a hemorrhagic event precluded their use.

Finally, this case illustrates the crucial value of OCT‐guided PCI. OCT enables precise lesion characterization, accurate stent sizing, assessment of stent expansion and apposition, and exclusion of edge dissections. In the present case, OCT findings were instrumental in guiding the decision to proceed with stent implantation, demonstrating an unstable plaque rupture, associated with a significant underlying stenosis, as well as a residual thin‐cap fibroatheroma (TCFA) with distal micro‐ruptures, reflecting ongoing plaque vulnerability [9]. Such high‐resolution guidance allows for a fully optimized intervention, minimizing procedural risks and ensuring optimal long‐term outcomes, a key consideration in young patients in whom procedural durability is essential. Beyond the setting of ACS, intracoronary imaging has become an essential tool in chronic coronary syndromes, particularly in the management of complex PCI such as calcified lesions [10], left main disease [11], and chronic total occlusions [12], as well as for the management of procedural complications [13].

This case highlights the pivotal role of intracoronary imaging in young patients presenting with ACS. Performing OCT after adequate thrombus resolution enables accurate diagnosis while guiding tailored management, ensuring the most appropriate and durable treatment for each patient.

## Author Contributions

**Guillaume Guebey:** writing – original draft. **Mathieu Chong:** writing – original draft. **Adelin Barrier:** supervision, validation. **Sarah Mauler‐Wittwer:** supervision, validation. **Stéphane Noble:** supervision, validation. **Quentin Liabot:** conceptualization, supervision, validation, writing – original draft, writing – review and editing.

## Funding

The authors have nothing to report.

## Consent

The authors confirm that the patient provided written informed consent for publication of this case report.

## Conflicts of Interest

The authors declare no conflicts of interest.

## Data Availability

All relevant data are included within the article.
